# Ghrelin, a novel therapy, corrects cytokine and NF-κB-AKT-MAPK network and mitigates intestinal injury induced by combined radiation and skin-wound trauma

**DOI:** 10.1186/s13578-020-00425-z

**Published:** 2020-05-12

**Authors:** Juliann G. Kiang, Joan T. Smith, Georgetta Cannon, Marsha N. Anderson, Connie Ho, Min Zhai, Wanchang Cui, Mang Xiao

**Affiliations:** 1 Scientific Research Department, Armed Forces Radiobiology Research Institute, Bethesda, MD 20814 USA; 2 Department of Pharmacology and Molecular Therapeutics, Uniformed Services, University of the Health Sciences, Bethesda, MD 20814 USA; 3 Department of Medicine, Uniformed Services, University of the Health Sciences, Bethesda, MD 20814 USA; 4 Department of Biochemistry, University of California, Berkeley, CA 94720 USA

**Keywords:** Ionizing radiation, Skin wound, Ghrelin, GI, NF-κB, AKT, ERK, JNK, MAPK, iNOS, BAX, Bcl-2, Caspase, Tight junction, Apoptosis, Bacteria

## Abstract

**Background:**

Compared to radiation injury alone (RI), radiation injury combined wound (CI) further enhances acute radiation syndrome and subsequently mortality. We previously reported that therapy with Ghrelin, the 28-amino-acid-peptide secreted from the stomach, significantly increased 30-day survival and mitigated hematopoietic death by enhancing and sustaining granulocyte-colony stimulating factor (G-CSF) and keratinocyte chemoattractant (KC) in the blood and bone marrow; increasing circulating white blood cell depletion; inhibiting splenocytopenia; and accelerating skin-wound healing on day 30 after CI. Herein, we aimed to study the efficacy of Ghrelin on intestinal injury at early time points after CI.

**Methods:**

B6D2F1/J female mice were exposed to ^60^Co-γ-photon radiation (9.5 Gy, 0.4 Gy/min, bilateral), followed by 15% total-body-surface-area skin wounds. Several endpoints were measured: at 4–5 h and on days 1, 3, 7, and 15.

**Results:**

Ghrelin therapy mitigated CI-induced increases in IL-1β, IL-6, IL-17A, IL-18, KC, and TNF-α in serum but sustained G-CSF, KC and MIP-1α increases in ileum. Histological analysis of ileum on day 15 showed that Ghrelin treatment mitigated ileum injury by increasing villus height, crypt depth and counts, as well as decreasing villus width and mucosal injury score. Ghrelin therapy increased AKT activation and ERK activation; suppressed JNK activation and caspase-3 activation in ileum; and reduced NF-κB, iNOS, BAX and Bcl-2 in ileum. This therapy recovered the tight junction protein and mitigated bacterial translocation and lipopolysaccharides levels. The results suggest that the capacity of Ghrelin therapy to reduce CI-induced ileum injury is mediated by a balanced NF-κB-AKT-MAPK network that leads to homeostasis of pro-inflammatory and anti-inflammatory cytokines.

**Conclusions:**

Our novel results are the first to suggest that Ghrelin therapy effectively decreases intestinal injury after CI.

## Background

Victims from radiation exposure accidents and nuclear weapon detonations experience radiation injury (RI), and often concurrently with physical trauma, such as wounds, burns, blast and/or hemorrhage—thus inducing, namely, combined injury (CI). CI was observed following the atomic bombings in Hiroshima and Nagasaki, Japan, as well as at the Chernobyl reactor meltdown. 60–70% of victims [1–4] and 10% of victims [5], respectively, received thermal burns alongside RI. Burns, wounds, infections, and hemorrhage typically increase mortality after otherwise non-lethal radiation doses observed in CI animal models, including mice [6–16], rats [17–19], guinea pigs [20], dogs [21, 22] and swine [23]. CI also delays wound closure times from normally 14–21 days after wounding without radiation to more than 30 days after irradiation [6, 9, 10, 24].

Altogether, CI leads to more severe outcomes than RI. CI has been observed to accelerate body-weight loss; amplify cytokine/chemokine imbalance and systemic bacterial infection [6, 16]; enhance leukocytopenia, thrombocytopenia, erythrocytopenia [7, 9, 10, 15], acute myelosuppression, immune system inhibition, fluid imbalance, macro- and microcirculation failure, massive cellular damage and disruption of vital organ functions. These subsequently led to multiple organ dysfunction (MOD) and multiple organ failure (MOF). Ultimately, death occurs as a result of CI [1, 20, 25, 26].

In our mouse model for RI and CI, the LD_50/30_ for RI is 9.65 Gy, whereas radiation-wound CI (R-W CI) is 8.95 Gy. The dose modifying factor (DMF) is 1.08 [6]. It is speculated that intervention of CI-induced aggravation of body-weight loss, cytokine imbalance or bacterial sepsis should improve the survival. Drugs or biologics such as pegylated G-CSF [9], Ciprofloxacin [10, 24], mesenchymal stem cells [27], and Ghrelin [28], prove to be effective for increasing 30-day survival.

The Ghrelin hormone is a hunger-stimulating peptide, containing 28 amino acids [29], produced mainly by P/D1 cells lining the fundus of the human stomach and by epsilon cells of the pancreas [30]. Ghrelin levels increase before meals and decrease after meals. Its counterpart hormone, leptin, is produced by adipose tissue [31].

Ghrelin potently stimulates growth hormone from the anterior pituitary gland [29], and it also activates the endothelial isoform of nitric oxide synthase (eNOS) in a pathway [32] that depends on the PI3K/Akt/eNOS/NO signaling pathway [33, 34]. Ghrelin binds on growth hormone secretagogue receptors that are coupled to G-proteins [29].

It has been reported that human Ghrelin decreases organ injury and increases survival by 30% above vehicle-treated mice after RI combined with severe sepsis in rats [18]. Kiang et al. [28] reported that Ghrelin therapy was efficacious in a mouse model of radiation combined with wounds or burns by increasing survival, body weight, wound healing, bone marrow cell counts, neutrophil recovery and platelet recovery on day 30 post-CI, as well as by inhibiting brain hemorrhage [35]. Ghrelin blocks NF-κB activation, decreases TNF-α and IL-6 concentrations in the lungs of septic rats, and inhibits nucleotide-binding oligomerization domain-containing protein 2 {NOD2, also known as caspase recruitment domain-containing protein 15 (CARD15) or inflammatory bowel disease protein 1 (IBD1)} [36]. NOD2 is important for apoptosis and NF-κB activation pathways [37]. Ghrelin inhibits IκB and increases Th1 cytokine and IL-17 secretion in primary T cells [38]. Furthermore, human Ghrelin mitigates jejunum injury and mortality after irradiation alone in rats [39]. We reported that Ghrelin mitigated CI-induced bone marrow damage by sustaining G-CSF and KC increases in bone marrow and circulation [40]. Other laboratories have demonstrated that Ghrelin suppresses inflammation and neuronal nitric oxide synthase [41]. However, Ghrelin’s effects on CI-induced ileum injury were unclear and possible underlying mechanisms were not delineated either. Because RI and CI prominently induce increased production of inflammatory cytokines/chemokines [6–8], the idea is that interventions mitigate inflammatory responses early on showing promise for improving ileum injury after CI. We, therefore, wanted to elucidate the dynamic changes in ileum cell death and its related signaling molecules. This report provides evidence from an experimental CI animal model, which was designed to demonstrate a kinetic change in Ghrelin effects at early time points after CI in ileum histopathology, circulating cytokines/chemokines, and apoptosis-related molecules including AKT-MAPK activation, NF-κB, and iNOS.

## Methods

### Experimental design

B6D2F1/J female mice were randomly divided into 8 groups: (1) sham vehicle, (2) wound vehicle, (3) RI vehicle, (4) CI vehicle, (5) sham ghrelin, (6) wound ghrelin, (7) RI ghrelin, and (8) CI ghrelin. Groups 2, 4, 6 and 8 received topical gentamicin cream; groups 1-8 were administered with oral levofloxacin. N = 6 mice per group per time point. Hematological analysis, spleen weights, splenocyte counts, sternum histology and femur bone marrow cell counts of surviving animals were performed at each specified time point. The AFRRI Institutional Animal Care and Use Committee reviewed and approved all animal procedures. Euthanasia was carried out in accordance with the recommendations and guidance of the American Veterinary Medical Association [42, 43].

### Animals

B6D2F1/J female mice (The Jackson Laboratory, Bar Harbor, ME) were maintained in a facility accredited by the Association for Assessment and Accreditation of Laboratory Animal Care International in plastic microisolator cages on hardwood chip bedding. Commercial rodent chow and acidified tap water were provided ad libitum at 12 to 20 weeks of age. Animal holding rooms were maintained at 21 °C ± 1 °C with 50% ± 10% relative humidity using at least 10 changes/h of 100% conditioned fresh air. A 12-h 0600 (light) to 1800 (dark) full-spectrum lighting cycle was used.

### Gamma irradiation

Mice were given 9.5 Gy [7] whole-body bilateral ^60^Co γ-photon radiation, delivered at a dose rate of 0.4 Gy/min, while held in vertically stacked, ventilated, four-compartment, acrylic plastic boxes that provided electron equilibrium during irradiation. Mice were not anesthetized in boxes where they could move freely. Empty compartments within the boxes were filled with 3-inch-long × 1-inch-diameter acrylic phantoms to ensure uniform electron scattering. The mapping of the radiation field was performed with alanine/EPR dosimetry [44] using standard alanine calibration sets from the U.S. National Institute of Standards and Technology and National Physical Laboratory of the United Kingdom. The mapping provided dose rates to water measured by alanine pellets placed in the hollowed core of the acrylic phantoms in each compartment of the mouse rack on the day of the mapping. The field was uniform within ± 1.8% over all the 120 compartments. The exposure time for each irradiation dose was determined from the mapping data; corrections for the ^60^Co decay and the small difference in the mass energy absorption coefficients for water and soft tissue were applied. The accuracy of the actual dose delivery was verified with an ionization chamber adjacent to the mouse rack, which had been calibrated in terms of dose to the mid-line soft tissue of mice.

### Skin injury

Skin surface injuries were performed on the shaved dorsal surface of mice. Animals receiving skin wounds were anesthetized by isoflurane inhalation. A 15% total body-surface-area skin wound was performed within 1 h after irradiation [6]. Briefly, an experimental wound was administered 19 ± 1.3 mm from the occipital bone and between the scapulae using a stainless- steel punch on a Teflon-covered board cleaned with 70% alcohol before each use. The anniculus carnosus muscle and overlying skin (23.5 ± 1.1 mm long and 14.9 ± 0.7 mm wide) were removed. All mice subjected to the skin injury were given 0.5 mL sterile 0.9% NaCl intraperitoneally (i.p.), which contained 150 mg/kg of acetaminophen (AmerisourceBergen, Glen Alen, Virginia), immediately after skin injury to alleviate pain. The sham group received the same handling procedure except administration of 0.5 ml sterile 0.9% NaCl containing 150 mg/kg of acetaminophen.

### Ghrelin

Ghrelin was purchased from Phoenix Pharmaceutical (Burlingame, CA). Three doses of 113 µg/kg were administered by lateral tail-vein injections [18] in a volume of 0.2 ml 24 h, 48 h and 72 h after RI or CI. The doses of Ghrelin were derived from the previous publications [18, 39]. The vehicle given to control mice was sterile 0.9% sodium chloride solution for injection, USP.

### Antimicrobial agents

Gentamicin sulfate cream, 0.1% (generic, E. Fougera and Co., Melville, N.Y., NDC 0168-007-15), was applied daily for 10 days to the skin injuries on days 1–10. Levofloxacin (LVX), (generic, Aurobindo Pharma, Ltd., Mahaboob Nagar, India, NDC 65862-537-50), 100 mg/kg in 0.2 ml/mouse, was administered *p.o*. daily for 14 days on days 3–16. Briefly, a 500-mg tablet was crushed by mortar and pestle. The LVX in the powder was dissolved in a volume of sterile water approximately one-third the total volume required to prepare the concentration needed for the average body mass of the mice to be treated. The mortar was rinsed with the remaining two-thirds volume of sterile water. The combined suspension was centrifuged to remove the particulate filler and the supernatant solution was passed through a 0.45-µm membrane filter into a sterile amber bottle, which was sealed with a sterile rubber stopper and stored at 4–8 °C [9].

### Histopathology assessment

Ileum specimens were collected from mice on day 15 (n = 6 mice per group). Specimens were rinsed in cold saline solution and immediately fixed in 10% phosphate-buffered formalin. The tissue was then embedded in paraffin, sectioned transversely and stained with H&E. The histology slides were scanned using Zeiss Axioscan.Z1. Then, villus height, villus width, crypt depth, and crypt numbers were counted and mucosal injury scores were assigned [45] using Zen 2 software (Zeiss Company, Thornwood, NY). To briefly summarize: grade 0 = normal mucosa; grade 1 = development of subepithelial spaces near the tips of the villi with capillary congestion; grade 2 = extension of the subepithelial space with moderate epithelial lifting from the lamina propria; grade 3 = significant epithelial lifting along the length of the villi with a few denuded villus tips; grade 4 = denuded villi with exposed lamina propria, dilated capillaries and reduced crypt death and counts; and grade 5 = disintegration of the lamina propria, hemorrhage, and ulceration.

### Cell apoptosis measurement

Ileum tissue slides were washed in three changes of xylene for 5 min each, in two changes of absolute ethanol for 5 min each, once in 95% ethanol and once in 70% ethanol for 3 min each and then once in PBS for 5 min. To observe cell apoptosis, slides were stained using ApopTag^®^ Plus Peroxidase in Situ Apoptosis Detection Kit (EMD Millipore Corp, Temecula, CA) following the manufacturer’s instructions.

### Measurement of cytokines/chemokines

Blood samples were collected at 4–5 h and on days 1, 3, 7, and 15 after RI or CI (N = 6 mice per group per time point) after RI or CI using BD Microtainers (Becton, Dickinson and Company, Franklin Lakes, NJ). Blood samples were placed at room temperature for 30 min and centrifuged at 9600×*g* for 10 min (Sovall Legend Micro 21 Centrifuge, Thermo Scientific). Then, serum was collected. Ileum samples were minced, blended with beads, homogenized with Bullet Blender Storm 24 (Averill Park, NY), and centrifuged at 9600x*g* for 10 min. The supernatants were collected. Cytokine/chemokine concentrations were measured and analyzed using the Bio-PlexTM Cytokine Assay (Bio-Rad; Hercules, CA) following the manufacturer’s directions. Briefly, serum samples and tissue lysates from each animal were diluted fourfold and examined. Data were analyzed using the LuminexH 100TM System (Luminex Corp.; Austin, TX) and quantified using MiraiBio MasterPlexH CT and QT Software (Hitachi Software Engineering America Ltd.; San Francisco, CA), and concentrations were expressed in pg/mL unless otherwise noted. The cytokines analyzed were IL-1α IL-1β, IL-2, IL-3, IL-4, IL-5, IL-6, IL-9, IL-10, IL-12(p40), IL-12(p70), IL-13, IL-15, IL-17A, IL-18, eotaxin, G-CSF, GM-CSF, IFN-c, KC, MCP-1, MIP-1a, MIP-1b, MIP-2, RANTES and TNF-a. Data were expressed as pg/mL in serum and pg/mg protein in tissues [6].

### Tissue lysates

Mice (N = 6 per group) were anesthetized by isoflurane followed by vertebrate dislocation at different time points after RI and CI for blood collection and tissue collection. Their ileum were collected. The ileum samples were mixed with Na^+^ Hanks’ solution containing 10 μl/ml protease inhibitor cocktail, 10 mM phosphatase 2 inhibitor, 10 mM phosphatase 3 inhibitor, 10 mM DTT, 5 mM EDTA and 10 mM PMSF, homogenized using Bullet Blender Homogenizer Storm (Next Advance, Averill Park, NY) for 4 min at speed 10 and centrifuged at 9000 xg for 10 min (Sorvall Legend Micro 21 Centrifuge, Thermo Electron Corp, Madison, WI). Supernatant fluids were conserved for protein determination and stored at −80 °C until use.

### Western blot

Total protein in the ileal lysates was determined with Bio-Rad reagent (Bio-Rad, Richmond, CA). Samples with 20 μg of protein in Na^+^ Hanks’ buffer containing 1% sodium dodecyl sulfate (SDS) and 1% 2-mercaptoethanol were resolved on SDS–polyacrylamide slab gels (Novex precast 4–20% gel, Invitrogen, Carlsbad, CA). After electrophoresis, proteins were blotted onto a polyvinylidene difluoride (PVDF) membrane (0.45 µm, Invitrogene) using a Tran-Blot Turbo System and the manufacturer’s protocol (Bio-Rad, Hercules, CA). The blot was then incubated for 90 min at room temperature with 5% non-fat dried milk in tris-buffered saline-0.5% tween20 (TBST, pH = 8.6) at room temperature. After blocking, the blot was incubated with a selected antibody against NF-κBp65 (catalog no. 8008), iNOS (catalog no. sc7271), BAX (catalog no. sc20067), Bcl-2 (catalog no. sc7382) (Santa Cruz Biotechnology, Dallas, TX), AKT (catalog no. ab941263), p-AKT (catalog no. ab179463), ERK1/2 (catalog no. ab115799), p-ERK1/2 (catalog no. ab50011), JNK (catalog no. ab179461). P-JNK (catalog no. ab124956), p38 (catalog no. ab31828), p-p38 (catalog no. ab195049) (ABCAM, Cambridge, MA), Claudin 2 (catalog no. 32-5600) (Invitrogen, Waltham, MA) and IgG (catalog no. HAF008) (R & D Systems, Minneapolis, MN) at an approximately final concentration of 1–2 μg/ml in TBST −5% milk. The blot was washed 3 times (10 min each) in TBST before incubating for 60 min at room temperature with a 1000X dilution of species-specific IgG peroxidase conjugate (Santa Cruz, CA) in TBST. The blot was washed 6 times (5 min each) in TBST before detection of the peroxidase activity using the Enhanced Chemiluminescence kit (Amersham Life Science Products, Arlington Height, IL). IgG levels were not altered by radiation and were used as a control for protein loading. Protein bands of interest were quantitated using the ImageJ program and normalized to IgG. Data were expressed as intensity ratio to IgG levels [6].

### Ghrelin measurement

Ghrelin in ileum lysate samples was measured using RayBio^®^ Human/Rat Ghrelin Immunoassay Kit (RayBiotech, Norcross, GA) according to the manufacturer’s protocol [46].

### Activated caspase-3 measurement

Activated caspase-3 protein levels were measured using the Quantikine ELISA kit according to the manufacturer’s protocol (R&D SYSTEM, Minneapolis, MN).

### Bacterial translocation

Bacterial translocation was determined as bacterial load in liver tissue and was quantified by real-time PCR using the 16S rRNA gene consensus sequence as described by Banerjee et al. [47]. The total load of bacteria in the liver was determined using primer sequences to amplify the highly conserved sequence for a broad species consensus. Livers were removed aseptically and stored at −80 °C until use. Bacterial translocation was quantified by real-time PCR. Briefly, liver tissue was homogenized in a MP FastPrep 24 Instrument (MP biomedicals, Irvine, CA) using Green Bead Lysis kits (Next Advance, Troy, NY). DNA was isolated from liver lysates using a DNA purification kit (Promega, Madison, WI). Bacterial DNA and liver genomic DNA concentration were quantified using Quant-iT™ PicoGreen™ dsDNA Assay Kit (Thermo Fisher Scientific, Waltham, MA). Serially diluted bacterial genomic DNA was used to generate the standard curve. Real-time PCR was performed using PowerUp SYBR green PCR master mix (Applied Biosystems, Foster City, CA) and 16S rRNA gene targeted primers, forward (5′-ACTCCTACGGGAGGCAGCAGT-3′) and reverse (5′-TATTACCGCGGCTGCTGGC-3′) in a QuantStudio 3 Realtime PCR System (Thermo Fisher Scientific, Waltham, MA). PCR-derived bacterial counts were expressed as nanogram bacterial DNA per gram mouse liver tissue.

### Lipopolysaccharides measurement

Lipopolysaccharides (LPS) levels were measured using LPS ELISA Kit (Antibodies-online.com, Catalog no. ABIN6574100) following the manufacturer’s protocol.

### Statistical analysis

Data are expressed as the mean ± s.e.m. For each experiment, 6 mice per group were tested on an individual basis. One-way ANOVA, two-way ANOVA, studentized-range test, and Student’s *t* test were used for comparison of groups, with 5% as a significant level.

## Results

### Ghrelin therapy inhibits circulating cytokines and chemokines after RI and CI

**Fig. 1:**
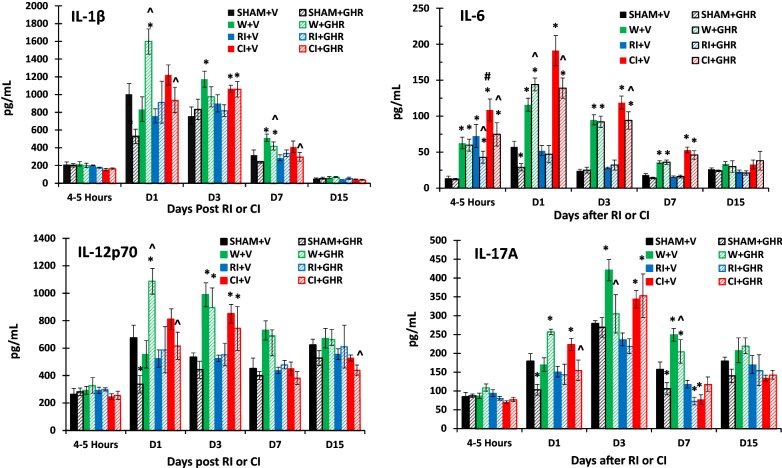
Effect of Ghrelin therapy on IL-1β, IL-6, IL-12p70 and IL-17A after RI and CI. Animals were irradiated alone or followed by wounding. Blood samples at different time points were collected for measuring IL-1β, IL-6, IL-12p70 and IL-17A concentrations in serum (N = 6 per group). The data presented are mean ± sem. *p < 0.05 vs. Sham vehicle group; ^p < 0.05 vs. respective vehicle group; #p < 0.05 vs. RI + Veh group. Veh or V: vehicle; Ghr or GHR: Ghrelin; *W* wounding; RI: 9.5 Gy; CI: 9.5 Gy + wounding

**Fig. 2:**
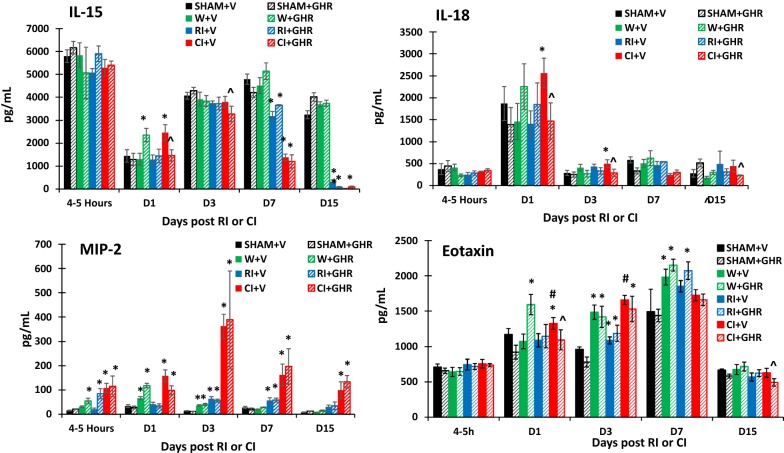
Effect of Ghrelin therapy on IL-15, IL-18, MIP-2 and Eotaxin after RI and CI. Animals were irradiated alone or followed by wounding. Blood samples at different time points were collected for measuring IL-15, IL-18, MIP-2 and Eotaxin A concentrations in serum (N = 6 per group). The data presented are mean ± sem. *p < 0.05 vs. Sham vehicle group; ^p < 0.05 vs. respective vehicle group; #p < 0.05 vs. RI + V group. *V* vehicle, *GHR* Ghrelin, *W* wounding; RI: 9.5 Gy; CI: 9.5 Gy + wounding

**Fig. 3:**
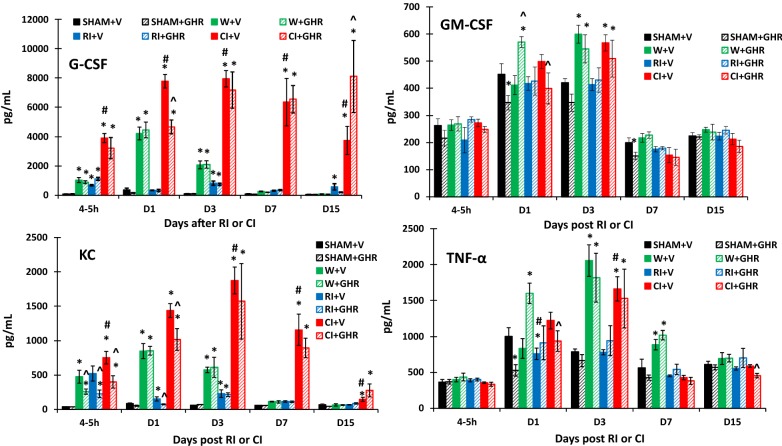
Effect of Ghrelin therapy on G-CSF, GM-CSF, KC and TNF-α after RI and CI. Animals were irradiated alone or followed by wounding. Blood samples at different time points were collected for measuring G-CSF, GM-CSF, KC and TNF-α concentrations in serum (N = 6 per group). The data presented are mean ± sem. *p < 0.05 vs. Sham vehicle group; ^p < 0.05 vs. respective vehicle group; #p < 0.05 vs. RI + V group. *V* vehicle, *GHR* Ghrelin, *W* wounding; RI: 9.5 Gy; CI: 9.5 Gy + wounding

It is evident that RI alone induced increases in circulatory cytokines/chemokines and CI further induced the increases [6]. Figures 1, 2 and 3 show no changes in IL-1β, IL-12p70, IL-15, IL-17A, IL-18, Eotaxin, GM-CSF and TNF-α 4–5 h after sham, wounding, RI and CI. However, wounding, RI, and CI increased IL-6, G-CSF, and KC. CI-induced increases were greater than RI-induced levels.

On day 1, wounding increased IL-6, G-CSF and KC; RI increased only KC, whereas CI increased IL-6, IL-17A, IL-15, IL-18, MIP-2. G-CSF and KC. Treatment with Ghrelin further increased IL-1β, IL-6, IL-12p70, IL-15, IL-17A, Eotaxin, GM-CSF, and TNF-α in wounded mice. In contrast, Ghrelin reduced RI-induced increases in KC and CI-induced increases in IL-1β, IL-6, IL-12p70, Il-15, IL-17A, IL-18, MIP-2, Eotaxin, G-CSF, GM-CSF, KC and TNF-α in blood.

On day 3, wounding and CI increased IL-1β, IL-6, IL-12p70, IL-17, Eotaxin, G-CSF, GM-CSF, KC, and TNF-α in blood, whereas RI increased only G-CSF and KC in blood. Treatment with Ghrelin did not alter wound- and RI-induced increases in cytokines mentioned above on this day, but attenuated CI-induced increases in IL-6, IL-15 and IL-18 in blood.

On day 7, wounding still increased IL-1β, IL-6, IL-12p70, IL-17A, Eotaxin, and TNF-α, RI only increased MIP-2. CI still increased IL-1β, IL-6, MIP-2, G-CSF and KC. Treatment with Ghrelin attenuated only IL-1β in blood of wounded and CI mice. CI-induced increases in IL-6, G-CSF and KC were not altered by Ghrelin.

On day 15, IL-1β, IL-6, IL-17A and GM-CSF returned to their baselines. Ghrelin treatment dropped IL-12p70, IL-18, Eotaxin and TNF-α below their baselines only in the blood of CI mice, while further increasing circulating MIP-2, G-CSF and KC in these CI mice.

### Ghrelin therapy decreases IL-18 levels after RI and CI in ileum

**Fig. 4:**
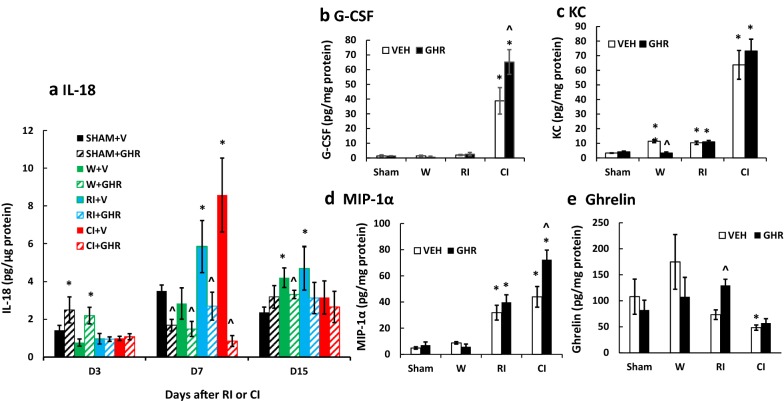
Effect of Ghrelin therapy on IL-18, G-CSF, KC and MIP-1α after RI and CI in ileum. Animals were irradiated alone or followed by wounding. Ileum samples at different time points were collected for measuring IL-18, G-CSF, KC and MIP-1α concentrations in ileum (N = 6 per group). The data presented are mean ± sem. *p < 0.05 vs. Sham vehicle group; ^p < 0.05 vs. respective vehicle group; #p < 0.05 vs. RI + V group. *V* vehicle, *GHR* Ghrelin, *W* wounding; RI: 9.5 Gy; CI: 9.5 Gy + wounding

Because RI- [48] and CI- [16] induced increases in IL-18 concentrations in the blood were significantly attenuated by Ghrelin, IL-18 concentrations in tissues were measured. Figure 4 shows the presence of IL-18 detected in ileum, a radiation-sensitive organ. Wounding did not alter IL-18 basal levels on day 3 and day 7 but significantly increased IL-18 on day 15. RI increased IL-18 on days 7 and 15; CI increased IL-18 only on day 7. Ghrelin therapy increased IL-18 in ileum of sham and wounded mice but not RI and CI mice on day 3. On day 7, Ghrelin therapy significantly attenuated IL-18 below the basal levels in ileum of sham, wounded, RI, and CI mice. On day 15, Ghrelin therapy reduced IL-18 concentrations in ileum of wounded and RI mice. IL-18 returned to basal levels in ileum of Ghrelin- or vehicle-treated CI mice.

### Ghrelin therapy increases and sustains G-CSF levels after CI, as well as KC and MIP-1α levels after RI and CI in ileum

On day 7, CI-induced increases in G-CSF and MIP-1α were further increased by Ghrelin (Fig. 4b, d). Increases in KC were also sustained in the presence of Ghrelin (Fig. 4c). The ghrelin levels in ileum lysate samples were also measured. As shown in Fig. 4e, RI tended to decrease ghrelin in ileum and Ghrelin therapy returned it to the basal level of sham animals. CI significantly decreased ghrelin levels in ileum, whereas the Ghrelin therapy recovered it to levels similar to those of the sham group.

### Ghrelin therapy recovers villus height, villus width, crypt depth and crypt count of ileum after RI and CI

**Fig. 5:**
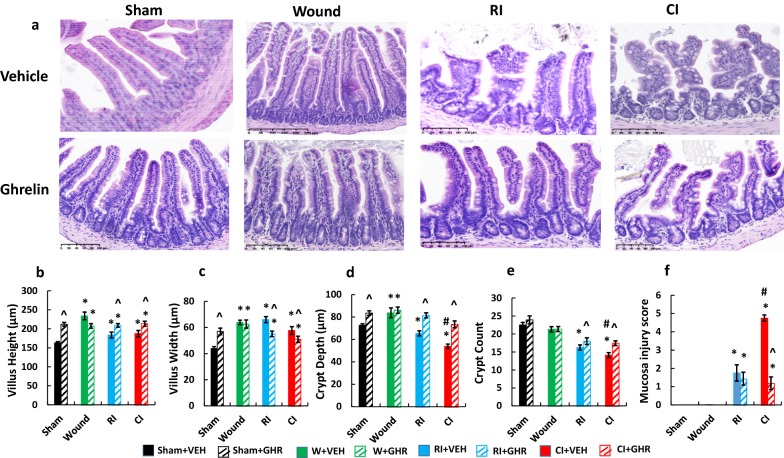
Ghrelin therapy mitigates ileum injury. Animals were irradiated alone or followed by wounding. Ileum samples on day 15 were collected for histology with H&E staining (N = 4 per group). Villus height, villus width, crypt depth and crypt counts were measured. Mucosal injury scores were assigned according to the criteria described in the Materials and Methods section above. The data presented are mean ± sem. *p < 0.05 vs. Sham vehicle group; ^p < 0.05 vs. respective VEH group; #p < 0.05 vs. RI + VEH group. *VEH* vehicle, *GHR* Ghrelin, *W* wounding; RI: 9.5 Gy; CI: 9.5 Gy + wounding

It is evident that RI and CI cause GI injury [1]. Ileum histology analysis was performed with H&E staining. Figure 5 shows that RI and CI injured the ileum morphology and Ghrelin therapy mitigated the injury (Fig. 5a). RI and CI increased the villus height (Fig. 5b) and width (Fig. 5c), as well as caused significantly shallower crypt depths (Fig. 5d) and lower crypt counts (Fig. 5e) compared to the sham group. Ghrelin therapy mitigated all of these changes (Fig. 5a–e). In addition, RI and CI increased mucosal injury scores, based on the criteria described in the Materials and Methods section above, that were reduced in CI mice after Ghrelin therapy (Fig. 5f).

### Ghrelin therapy decreases cell apoptosis and caspase-3 activation in ileum after RI and CI

**Fig. 6:**
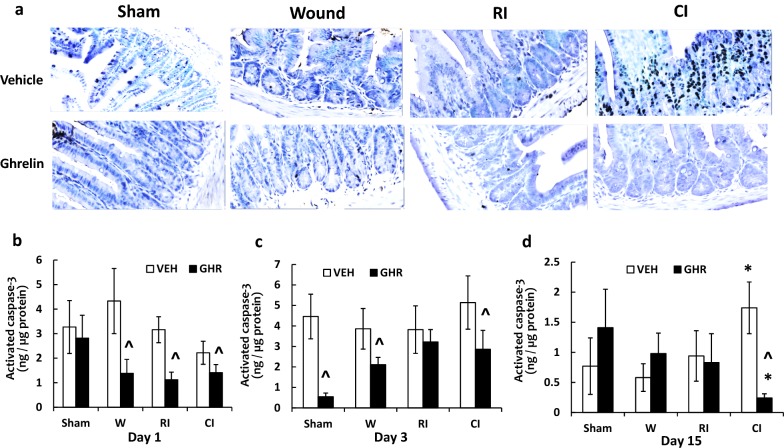
Ghrelin therapy decreases cell apoptosis and caspase-3 activation in ileum. Animals were irradiated alone or followed by wounding. A Slides from ileum samples from day 15 were stained for apoptosis assessment. The representative slides for each group are presented. B The apoptotic cells per crypt were counted. C-E Ileum samples at different time points were collected for measuring caspase-3 activation (N = 6 per group). The data presented are mean ± sem. *p < 0.05 vs. Sham VEH group; ^p < 0.05 vs. respective VEH group. *VEH* vehicle, *GHR* Ghrelin, *W* wounding; RI: 9.5 Gy; CI: 9.5 Gy + wounding

It is evident that RI [49] and CI [16] increase caspase-3 activation, which generally leads to cell apoptosis [1, 50]. Therefore, we performed an apoptosis assay to compare apoptotic cell death. In Fig. 6a, b. RI on day 15 significantly induced apoptotic death in crypts and CI further increased the number of cell death. However, Ghrelin therapy mitigated the cell death in CI ileum samples.

We also measured caspase-3 levels in ileum in order to verify whether cell apoptosis was involved in RI- and CI-induced ileum damage. We demonstrate that on day 1, caspase-3 activation was unchanged by wounding, RI and CI with vehicle treatment, compared to sham mice. Treatment with Ghrelin, however, significantly reduced caspase-3 activation in these wounded, RI and CI mice (Fig. 6c). On day 3, similar observations were found in ileum samples of vehicle-treated mice. Ghrelin significantly reduced caspase-3 levels in sham, wounded, and CI mice (Fig. 6d). On day 15, CI but not wounding and RI remarkably increased caspase-3 activation, but the increase was able to be inhibited by Ghrelin (Fig. 6e).

### Ghrelin increases AKT and ERK activation after CI and decreases JNK activation after wounding and RI

**Fig. 7:**
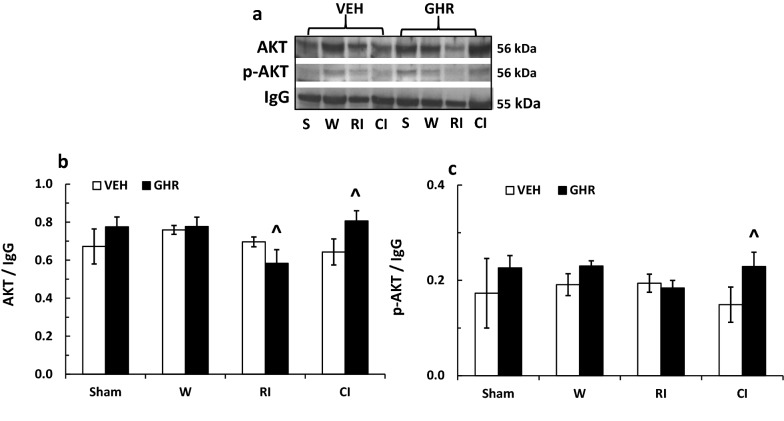
Ghrelin therapy increases AKT activation in ileum. Animals were irradiated alone or followed by wounding. Ileum samples collected on day 3 were analyzed for AKT activation (N = 4 per group). The data presented are mean ± sem. ^p < 0.05 vs. respective VEH group. *VEH* vehicle, *GHR* Ghrelin, *W* wounding; RI: 9.5 Gy; CI: 9.5 Gy + wounding

RI and CI alter AKT and MAPK activation [16] which regulate cell apoptosis [1, 51]. To evaluate whether Ghrelin altered their RI- and CI-induced changes, we measured them using Western blotting analysis. Figure 7 shows that AKT and phosphorylated AKT in ileum samples of vehicle-treated sham, wounded, RI, and CI mice on day 3 were similar, while AKT was reduced in Ghrelin-treated RI mice and increased in Ghrelin-treated CI mice (Fig. 7b). AKT phosphorylation was increased as well in CI mice treated with Ghrelin (Fig. 7c).

**Fig. 8:**
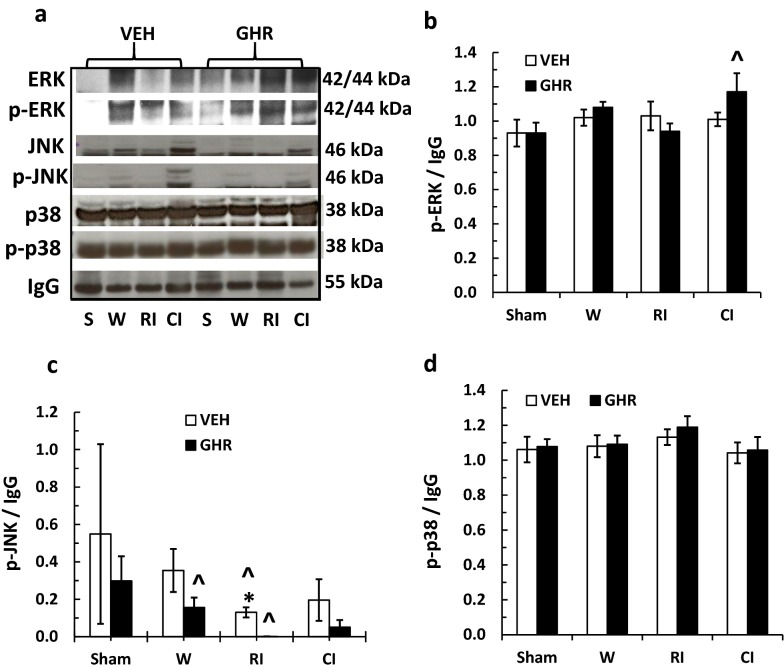
Ghrelin therapy increases ERK activation and decreases JNK activation in ileum. Animals were irradiated alone or followed by wounding. Ileum samples collected on day 3 were analyzed for AKT activation (N = 4 per group). The data presented are mean ± sem. ^p < 0.05 vs. respective VEH group. *VEH* vehicle, *GHR* Ghrelin, *W* wounding; RI: 9.5 Gy; CI: 9.5 Gy + wounding

Figure 8 depicts that wounding, RI, and CI did not alter the basal levels of ERK phosphorylation (p-ERK), whereas Ghrelin increased p-ERK after CI. RI decreased JNK phosphorylation (p-JNK), but Ghrelin significantly decreased p-JNK in wounded and RI mice. Phosphorylated p38 was not altered in wounded, RI, and CI mice treated with vehicle or Ghrelin.

### Ghrelin decreases NF-κB, iNOS, BAX and Bcl-2 after CI

**Fig. 9:**
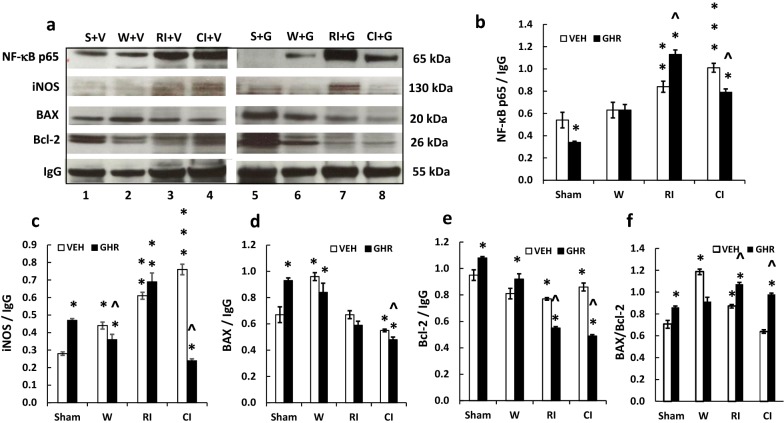
Ghrelin therapy decreases NF-κB, iNOS, BAX and Bcl-2 in ileum. Animals were irradiated alone or followed by wounding. Ileum samples collected on day 3 were for analyzed for NF-κB, iNOS, BAX and Bcl-2 (N = 4 per group). The data presented are mean ± sem. *p < 0.05 vs. sham vehicle group; **p < 0.05 vs. sham vehicle and wound vehicle groups; ***p < 0.05 vs. sham vehicle, wound vehicle and RI vehicle groups; ^p < 0.05 vs. respective VEH group. *VEH* vehicle, *GHR* Ghrelin, *W* wounding; RI: 9.5 Gy; CI: 9.5 Gy + wounding

Cell apoptosis is known to be initiated by increases in iNOS [1, 49, 50], which is transcribed by NF-κB [6]. Therefore, we first investigated NF-κB in ileum lysate samples on day 3. In vehicle-treated mice, wounding did not alter the basal expression of NF-κB as compared to the sham group. However, RI drastically increased NF-κB, while CI further increased it. Ghrelin reduced the levels of this transcription factor in sham and CI mice, compared to their respective vehicle groups. However, Ghrelin therapy increased additional NF-κB in RI mice (Fig. 9a, b). Then, we measured iNOS (Fig. 9a, c). In vehicle-treated mice, wounding altered the basal expression of iNOS compared to the sham group. Nonetheless, RI significantly increased iNOS protein and CI increased even more iNOS than RI. Ghrelin therapy increased iNOS in sham mice and reduced iNOS protein in wounded mice and CI mice but not in RI mice.

Ghrelin was found to reduce BAX (a pro-apoptotic protein) and increase Bcl-2 (an anti-apoptotic protein) in a chronic liver injury model [28]. We, therefore, measured BAX and Bcl-2 in ileum on day 3 after RI and CI. Wounding significantly increased BAX, but CI significantly decreased BAX. Ghrelin therapy significantly increased BAX in sham samples but significantly decreased BAX in wounding samples and CI samples, whereas it did not alter RI-induced decreases in BAX (Fig. 9a, d).

In Fig. 9a, there were 2 Bcl-2 bands found very close to each other at 26 kDa. We assumed the lower band being unphosphorylated Bcl-2 and the upper band being phosphorylated Bcl-2. Both bands were altered together. Therefore, we measured two bands together with ImageJ. Bcl-2 was decreased in wounding samples (lane 2), RI samples (land 3) and CI samples (lane 4) on day 3. Ghrelin therapy significantly increased both unphosphorylated and phosphorylated Bcl-2 bands in sham samples (lane 5), but significantly decreased both Bcl-2 bands in RI samples (lane 7) and CI samples [(lane 8) Fig. 9e]. The ratio of BAX to Bcl-2 (total of unphosphorylated band and phosphorylated band) was increased in sham, RI and CI samples while it was decreased in wounded samples (Fig. 9f).

### Ghrelin increases tight junction protein after CI

**Fig. 10:**
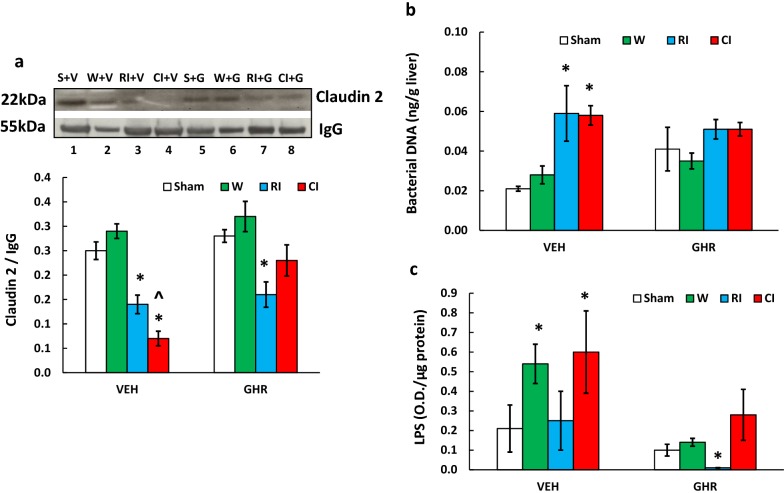
Ghrelin therapy increases tight junction protein in ileum and mitigates bacterial translocation and lipopolysaccharides (LPS) levels. **a** Animals were irradiated alone or irradiated followed by wounding. Ileum lysate samples collected on day 7 were used to detect claudin 2, a biomarker for the tight junction (N = 4 per group). **b** The liver lysate samples (100 mg per sample) from each animal (N = 6) were used for measuring bacterial DNA, presented as ng/g liver. **c** The liver lysate samples from each animal (N = 6) were used for measuring LPS levels using the LPS ELISA Kit, presented as O.D./µg protein. The data presented are mean ± sem. *p < 0.05 vs. RI + VEH. *W* wounding; RI: 9.5 Gy; CI: 9.5 Gy + wounding; *VEH* vehicle, *GHR* Ghrelin

Functional assays of small intestinal injury include permeability [52]. Radiation is known to decrease tight junction protein in small intestine resulting in leakage [47]. Therefore, tight junction biomarker claudin 2 was measured in ileum samples on day 7 using Western blot analysis. Figure 10a depicts that RI significantly decreased claudin 2 by 44% (p < 0.05), while CI further decreased it by 72% (p < 0.005) compared to the basal level of sham samples. Ghrelin therapy recovered claudin 2 to the basal level in CI samples, whereas it remained low in the RI samples.

### Ghrelin attenuates bacterial translocation after CI

Decreases in the tight junction in ileum allow passage of bacteria present in the lumen into the blood stream, and subsequently translocation to tissues that should be sterile [39, 47]. In the vehicle-treated animals, 16S rRNA gene, a measure of bacterial counts, was significantly risen by 190% (p < 0.05 vs. Sham + VEH) and 180% (p < 0.05 vs. Sham + VEH), respectively. Ghrelin therapy increased the basal level, but RI and CI only increased the bacterial counts by 24.7% (p > 0.05 vs. Sham + GHR) and 24.3% (p > 0.05 vs. Sham + GHR), respectively (Fig. 10b).

### Ghrelin attenuates lipopolysaccharides level after CI

LPS levels represents the presence of Gram-negative bacteria. As shown in Fig. 10c, in the vehicle-treated animals, LPS levels in liver samples of wounded mice and CI mice on day 7 were significantly increased by 257% (p < 0.05 vs. Sham + VEH) and 279% (p < 0.05 vs. Sham + VEH), respectively, whereas. RI did not significantly increase it (Fig. 10c). Ghrelin therapy effectively mitigated these LPS increases in RI and CI mice.

## Discussion

Our novel results are the first to show that Ghrelin therapy mitigates ileum injury caused by CI. Therapy with Ghrelin, a stomach-derived peptide with a half-life of approximately 31 min in plasma [15, 44], significantly increased and sustained G-CSF levels after CI. Ghrelin therapy also led to KC and MIP-1α increases after RI and CI, in addition to reduce IL-18 in RI and CI mice. Ghrelin therapy ameliorated ileum injury, likely due to its ability to alter cytokines and chemokines in blood and ileum.

The circulating G-CSF increase significantly contributed to ileum, spleen, kidney, bone marrow and likely other untested organs [1]. In the bone marrow, it is known to stimulate myeloid progenitors for neutrophil proliferation and maturation [1]. Herein, we also found that, in ileum, CI significantly increased G-CSF with Ghrelin therapy further increasing it. The sustaining G-CSF in blood and its further increases in ileum probably provided significantly therapeutic effects to ileum. It was reported that, in mice, heart diabetes increased microRNA-34a levels in cardiomyocytes which then inhibited Bcl-2 levels and increased apoptosis. Increases in G-CSF inhibited microRNA-34a and reduced cell apoptosis in the heart [53]. We also reported that pegylated G-CSF combined with a thrombopoietin receptor agonist effectively mitigated ileum injury [45]. In this report, decreases in BAX and Bcl-2 levels were found in ileum samples of RI and CI mice and Ghrelin therapy further decreased BAX in CI mice and Bcl-2 in RI and CI mice. The results agree with the observation found in a chronic liver injury model [32]. In contrast to the hearts of diabetic mice [53], we have detected RI and CI-induced increases in miR-34a (16), but Ghrelin therapy failed to inhibit these increases (Kiang et al., unpublished data). Decreases in Bcl-2 are indeed unfavorable in this case. Instead, decreases in BAX are beneficial and likely contribute to the mitigation of ileum injury [54].

CI significantly increased KC in blood and ileum. Ghrelin therapy maintained its levels above basal levels. Mainly produced by macrophages, neutrophils and epithelial cells [55, 56], KC has been shown to be critical for wound healing. Ghrelin therapy recovered bone marrow cellularity on days 1, 7, and 15 and circulating neutrophils on days 3 and 15 [28]. Therefore, KC attracting neutrophils to the wound area for wound repair becomes very significant. In KC receptor knockout mice, wound healing was significantly delayed compared to wild type mice [57], suggesting no involvement with neutrophils. Therefore, maintenance of high levels of KC is significantly beneficial towards repairing ileum injury.

CI increased IL-18 in blood and ileum, maintaining consistency with our previous observations [6, 39]. Ghrelin therapy significantly decreased IL-18 in blood and ileum. Mainly produced by macrophages, epithelial cells, and endothelial cells, IL-18 upregulates IFN-r and induces inflammation. It is associated with Alzheimer’s disease and diabetic nephropathy [58, 59]. Therefore, Ghrelin inhibition of IL-18 was desired in this case. IL-18 binding protein has been shown to increase mouse survival after lethal irradiation [60]. There is no report yet suggesting treatment with IL-18 binding protein enables to improve survival after CI. However, Ghrelin therapy further increased MIP-1α increases in ileum after RI and CI. MIP-1α is known to promote homeostasis [61].

The classical histological end point in mice is the number of regenerating crypts measured in the small intestine at 3.5 days after RI [52]. Our previous publications indicated that the morphology changes in small intestine of B6D2F1 mice exposed to either RI or CI could not be clearly distinguished at 3.5 days. However, differences began to show at 7 days, with clear separation by days 15 and 30, in which CI induced more detrimental effects than RI [1]. Therefore, we adopted ileum tissues on day 15 for studying the histological end point. Once again, RI and CI resulted in fewer crypt counts and smaller crypts in ileum, while Ghrelin therapy enabled the mitigation of the crypt loss (Fig. 5a, d, e). The observations were consistent with those found in other laboratories [39].

Caspase-3 is a critical protease in caspase-dependent apoptosis [62, 63] that increases in ileum following irradiation and combined injury [6, 16]. Herein, RI and CI increased caspase-3 activation, which agrees with our previous observations [6, 16]. Increases in capase-3 activation above basal levels were observed on day 15, suggesting that apoptosis occurred later. This observation was reinforced with data obtained from apoptosis assay in situ (Fig. 6a, b).

Ghrelin, in general, binds to GHSR-1α ghrelin receptors, coupling to G proteins. Ghrelin may have acted via mediation of the AKT/NOS/NO signal pathway [34] to ameliorate ileum injury. Ghrelin increased AKT phosphorylation and decreased JNK/MAPK phosphorylation in ileum of CI mice. In this strain of mice, CI did not change the basal levels of AKT and MAPK activation in ileum, but in the brain, CI decreased AKT, ERK/MAPK and JNK/MAPK activation [64]. This is inconsistent with observations found in CD2F1 mice, in which CI significantly increased ERK/MAPK and p38/MAPK activation in ileum [16]. Therefore, it is worthwhile to bear in mind that different strains of mice may respond to CI differently. Likewise, even within the same strain of mice, different organs may manifest different responses to CI. However, taken together, the data purport that resuming AKT activation by any means is critical for organ recovery and survival improvement [1, 51].

AKT activation triggers cell survival by regulating NF-κB [6, 51]. Although it was reported that NF-κB was required for AKT activation and that it resided upstream of AKT activation [65], it is evident that AKT activation was required for stimulating NF-κB [66–68]. Herein, we observed that CI did not alter the basal levels of AKT activation, but increased NF-κB p65, the transcription factor for IL-1, IL-8, IL-18, IL-33, TNF and Trail gene expression [51]. However, Ghrelin therapy increased AKT activation while reducing NF-κB. The latter is a transcription factor for iNOS expression as well, thereby leading to reduction by Ghrelin therapy. INOS causes apoptosis through activation of the intrinsic pathway involving apoptosome formation and caspase-3 activation [49].

Ghrelin therapy significantly diminished BAX in CI mice, which is desirable as a therapy. BAX is known to be regulated by the free p53 which is not conjugated by mdm2 [1]. The possibility of Ghrelin therapy to increase MDM2 (that can be stimulated by AKT activation) to conjugate with p53 [64], thus, leading to lower p53 in ileum cannot be excluded. Unlike BAX, Ghrelin therapy also attenuated Bcl-2, which is not beneficial as a therapy. Bcl-2 in cultured cells [69] and in C57BL/6 mice, Fabp/-Bcl-2 mice [70] and Sprague–Dawley rats [71] manifested one band using the immunoblotting technique. However, in our B6D2F1 mice, the ileum samples exhibited two bands closed to each other, suggesting presence of unphosphorylated Bcl-2 (lower band) and phosphorylated Bcl-2 (upper band). Moreover, two bands were altered together. Therefore, the Ghrelin inhibition on Bcl-2 in RI mice and CI mice needs to be further explored.

Ghrelin has been demonstrated as a countermeasure against radiation combined with sepsis [18]. Our data in CI mice (Fig. 10) are in agreement with the observation in RI rats published by Wang and colleagues [39]. We found Ghrelin therapy effectively recovered the tight junction of ileum in CI mice but not in RI mice. The discrepancy is due to (1) the female mice we studied vs. the male rats they studied and (2) mouse Ghrelin we used vs. human Ghrelin they used. It was suggested that the effect on rats was based on complex neurogenic effects of this peptide, involving with activation of the cholinergic pathway, inhibition of the sympathetic nervous system (SNS), and down-regulation of proinflammatory cytokines [41, 72, 73]. Therefore, Ghrelin′s beneficial effects following irradiation combined with sepsis may have been correlated with the rebalance of dysregulated sympathetic and parasympathetic (PNS) nervous systems [72]. It is possible that ghrelin-induced improvement of survival in our CI model is mediated by the rebalance of cytokines, SNS, and PNS. This hypothesis requires confirmation.

**Fig. 11:**
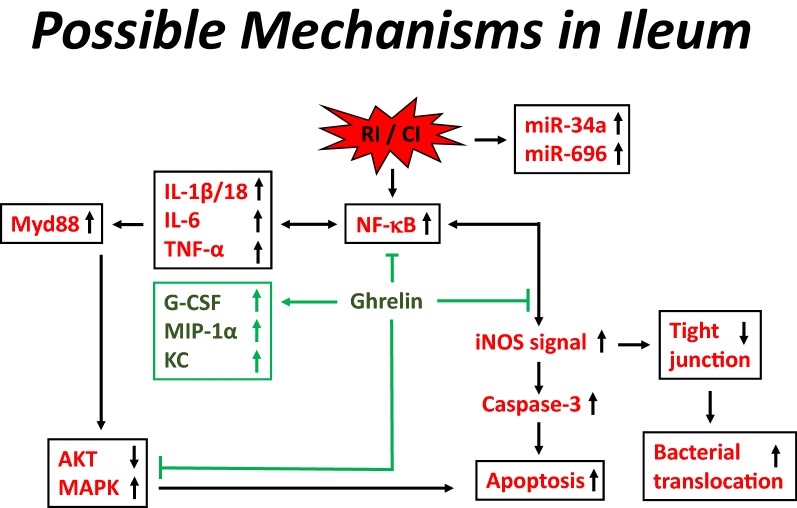
Possible mechanisms of Ghrelin therapy in mitigating ileum injury. RI and CI increase miR-34a and miR-696, as well as NF-κB. Increases in NF-κB expression trigger cytokines and chemokines in ileum. Increases in IL-1β/18, IL-6, and TNF-α stimulate Myd88, which reduces AKT activation and elevates MAPK activation, thereby increasing apoptosis. On the other hand, increases in NF-κB transcribe iNOS that triggers caspase-3 activation, also resulting in apoptosis. Ghrelin therapy inhibits NF-κB, reinforces RI/CI-induced increases in G-CSF, MIP-2 and KC and promotes tissue repair. This therapy also increases AKT activation which promotes cell survival

Ghrelin sustained G-CSF, KC and MIP-1α levels while decreasing IL-18 in ileum (Fig. 4), thereby promoting cell survival and repair of ileum injury. Ghrelin administration accelerated body weight recovery and found no edemas [35]. Figure 11 shows the possible mechanisms of RI and CI increasing miR-34a and miR-696, as well as NF-κB. Increases in NF-κB expression trigger cytokines and chemokines in ileum. Increases in IL-1β/18, IL-6, and TNF-α stimulate Myd88 which reduces AKT activation and elevates MAPK activation, leading to increases in apoptosis. On the other hand, increases in NF-κB transcribe iNOS which triggers caspase-3 activation resulting in apoptosis (Fig. 6). Ghrelin therapy inhibits NF-κB, reinforces RI/CI-induced increases in G-CSF, MIP-1α and KC and promotes tissue repair homeostasis. This therapy also increases AKT activation, recovering the ileum tight junction (Fig. 10a) while mitigating bacterial translocation (Fig. 10b) and promoting cell survival. Moreover, increased LPS levels in wounded and CI mice but not RI mice (Fig. 10c) suggest that Gram-negative bacteria probably mainly came from wound areas. The observation is consistent with the previous report [74].

It is not clear why Ghrelin therapy was effective in inhibiting NF-κB and iNOS in CI mice but not in RI mice. The results suggest that NF-κB and iNOS play important roles in causing ileum injury in CI mice. In contrast, our data showed that Ghrelin therapy decreased JNK activation in RI mice but not in CI mice, suggesting JNK probably plays a significant role in RI mice (Fig. 8c). It warrants further exploration in understanding the differential effects of Ghrelin therapy after RI and CI.

Ghrelin therapy is effective for increasing survival in rats exposed to radiation alone [39] or radiation combined with sepsis [18] and in mice exposed to radiation followed by skin wounding or burning [28]. This improvement is attributed, at least in part, by mitigation of bone marrow injury [40] and intestinal injury, as presented in this report. To further confirm the contribution of Ghrelin therapy to mitigating intestinal injury, it is ideal to have a mouse model with bone marrow transplantation [11] or partial shield of bone marrow to preserve bone marrow [75] first then followed by local irradiation to the abdomen focusing on intestine. Then, the intestinal injuries can be investigated thoroughly. With it, potential medical countermeasures to treat intestinal injuries can be identified, which is this study’s ultimate goal.

## Conclusion

Skin wounds enhances CI-induced ileum injury, as indicated by increases in villus edema, decreases in crypt depth and crypt counts, as well as increases in mucosal injury scores, all of which are mitigated by Ghrelin therapy. This alleviation was confirmed by significant increases in G-CSF, KC and MIP-1α, decreases in IL-1β, IL-6, IL-18, TNF-α and caspase-3 activation, increases in AKT and ERK activation and decreases in iNOS and BAX in ileum of CI mice through NF-κB inhibition. The recovery of ileum function is manifested by recovery of tight junction and reduction of bacterial translocation. These results demonstrate Ghrelin’s efficacy as a potential radiomitigator and radiotherapy agent for treating CI.

## Data Availability

All relevant data are within this published paper.

## Abbreviations

AFRRI: Armed forces radiobiology research institute
AKT: Protein kinase B (specific for serine/threonine phosphorylation)
BAX: Bcl-2-associated X protein
Bcl-2: B-cell lymphoma 2
CI: Combined injury
ERK: Extracellular signal–regulated kinase
G-CSF: Granulocyte-colony stimulating factor
GHSR: Growth hormone secretagogue receptor
iNOS: Inducible nitric oxide synthase
i.v.: Intravenous
JNK: c-Jun N-terminal kinase
KC: Keratinocyte chemoattractant
MAPK: Mitogen-activated protein kinase
MDM2: Mouse double minute 2 homolog
p38: Protein 38
p53: Protein 53
p.o.: *per os*
RCI: Radiation combined injury
RI: Radiation injury
s.c.: Subcutaneous
TBSA: Total body surface area
VSD: Veterinary science department
W: Wound
